# Elimination of persistent vaccine bacteria of *Salmonella enterica* serovar Typhimurium in the guts of immunized mice by inducible expression of truncated YncE

**DOI:** 10.1371/journal.pone.0179649

**Published:** 2017-06-19

**Authors:** Yiran Wang, Jianhua Li, Kun Xiong, Zhijin Chen, Chunping Zheng, Yong Tan, Yanguang Cong

**Affiliations:** 1 Department of Microbiology, Third Military Medical University, Chongqing, China; 2 The Orthopaedic Center of PLA, 88th Hospital of PLA, Tai’an, Shandong Province, China; Robert Koch Institute, GERMANY

## Abstract

Orally administered vaccine bacteria usually persist for a period of time in the intestinal tracts of immunized individuals, and are excreted in feces to the environment resulting in a potential biosafety issue. The releasing risk can be minimized by immediate elimination of the persistent vaccine bacteria once adequate protective immune responses have been elicited by the vaccine bacteria. In a previous study, inducible expression of truncated *yncE* gene (yncE*) was found lethal to host bacteria. This feature has an application potential in biosafety control. Here, we assessed the efficacy of YncE* in eliminating an attenuated strain of *Salmonella enterica* serovar Typhimurium in a mouse model. To this end, a pBAD-derived plasmid containing yncE* under the control of the Ara promoter was transformed into a Δ*phoPQ* mutant of *S*. Typhimurium. Our data show that the induced expression of yncE* in the presence of arabinose eliminated the vaccine bacteria both in vitro and in vivo. BALB/c mice with or without streptomycin-pretreatment were used to assess the efficacy of YncE* in vivo. Oral administration of 500 μl of 20% arabinose at 24 h postvaccination removed the vaccine bacteria from the guts of the tested mice without streptomycin-pretreatment. For streptomycin-pretreated mice, which were colonized with higher levels of *Salmonella*, an additional gavage of arabinose was required to completely eliminate the vaccine bacteria in the guts of the tested mice. The orally administered arabinose did not affect the persistence of bacteria that had penetrated the intestinal mucosa of the immunized mice. Furthermore, there was no significant difference in the protection rate between the routine immunization and the immunization with the arabinose treatment. The results indicate that the yncE* element improves the biosafety of the bacterial vaccine, and can be taken in consideration in future design of live bacterial vaccines.

## Introduction

Live bacterial vaccines possess multiple advantages including simple manufacture, high acceptance, low rates of side-effects, and a convenient vaccination process which triggers systemic immune and mucosal immune responses [1]. Additionally, the attenuated vaccines can be used as vectors for vaccines against other infections or antitumor agents [2, 3]. Nevertheless, although the excellent safety of the licensed live vaccines had been demonstrated in a number of clinical investigations, there are health and biosafety concerns regarding the release of genetically modified bacteria into the environment [4, 5]. For example, orally administered vaccine bacteria usually persist in the guts of immunized individuals after vaccination, and display fecal shedding for a period of time, from several days to several weeks [6–10].

To minimize the release risk, the fecal shedding is ideally terminated as soon as possible once the protective immunization has been established in the immunized individual. In an attempt to shorten the shedding duration, Ghany et al. identified several mutations in *Salmonella enterica* serovar Typhimurium, including *hdA*, *misL*, and *ratB*, that reduced shedding in mice without alteration in immunogenicity [11]. Another strategy is to built a conditional lethal system in recombinant bacteria [12]. Many suicidal genetic elements have been employed in these systems, including *gef* and *relF* of *Escherichia coli* [12–14], the *E* gene of bacteriophage ΦX174 [15], the *hok* gene of the plasmid R1 [16], colicin E3-encoding gene [17], etc. These conditional lethal genetic elements harbored in the vaccine bacteria can be triggered by environmental circumstances, and specifically kill the host bacteria without interfering with the normal flora.

In a previous study, we found that the inducible expression of truncated *yncE* gene (yncE*) impaired bacterial membrane structure, leading to lysis of host bacteria [18]. The *yncE* gene is conserved in *Enterobacteriaceae* with unknown physiological function [19]. The YncE protein is characterized by a typical seven-bladed β-propeller structure [19, 20], and had been identified as a prominent antigen expressed uniquely in patients who carried *S*. Typhi in their biliary tracts, suggesting YncE might contribute to bacterial survival in the biliary tract [21]. YncE is also a conserved immunogenic antigen of *E*. *coli* able to protect against acute systemic infection in mice [22]. In the present study, we explored whether or not yncE* can be used to clear bacterial cells of an attenuated *S*. Typhimurium strain administered in a mouse model. Moreover, the impact of the elimination treatment on the efficacy of the vaccine bacteria was also assessed.

## Materials and methods

### Ethics statement

The protocol of animal experiment was reviewed and approved by the laboratory animal welfare and ethics committee of Third Military Medical University. The animal experiments were performed according to the Guideline on the Humane Treatment of Laboratory Animals of the Ministry of Science and Technology of China.

### Strains, plasmids and medium

*S*. Typhimurium CMCC50115 was obtained from the National Center for Medical Culture Collections (Beijing, China), and was used as a wild-type strain. *E*. *coli* S17-1/λ_pir_ was a gift from Dr. Victor de Lorenzo of the Centro Nacional de Biotecnologia CSIC, Spain. A suicide vector pYG4 described previously was used for construction of deletion mutant [23]. Recombinant plasmid pBAD24-yncE* harboring the truncated *yncE* gene (the 5’-terminal 408 bp) under the control of the Ara promoter was constructed previously [18]. Unless otherwise noted, bacteria were grown in an animal product free medium composed of 1% vegetable peptone, 0.5% yeast extract and 1% NaCl. When required, kanamycin and ampicillin were used at 50 μg/ml and 100 μg/ml, respectively.

### Construction of *phoPQ*-deletion mutant of *S*. Typhimurium

Deletion of the *phoPQ* locus in *S*. Typhimurium was performed via homologous recombination as described previously [23, 24]. Briefly, the upstream and downstream fragments of *phoPQ* were PCR-amplified from the genomic DNA of *S*. Typhimurium with primers P1 (cccgggcccccttacaccacccagattga) and P2 (cccgttataaatttggagtgtgaaggttattgcgtgctcttctcccttgtgttaac), as well as primers P3 (ccccacgcaataaccttcacactccaaatttataacacatttctgtgcgttcttcc) and P4 (cccgagctcccggatcgctgtagtatgta), and spliced by overlap PCR with primers P1 and P4. After restriction enzyme digestion by ApaI and SacI, the fused fragment was inserted into pYG4 pretreated with the same restriction enzymes, resulting in pYG4-ΔphoPQ. The recombinant pYG4-ΔphoPQ was transformed into competent cells of *S*. Typhimurium by electroporation, and screened for chromosomal integration of the plasmid with kanamycin selection. After an overnight-incubation in antibiotic-free medium, plasmid-excised strains were reversely selected on agar supplemented with 5% sucrose. The Δ*phoPQ* strain was then screened and identified by PCR with primers P5 (gaagaggtcatcaaactcac) and P6 (catcgtaaatcagcgtcatg), which amplify an internal fragment of *phoPQ*.

### Determination of 50% lethal dose (LD_50_)

Fresh cultures of the wild-type strain and the Δ*phoPQ* mutant of *S*. Typhimurium were washed and resuspended in phosphate-buffered saline (PBS) to get ten-fold dilutions (2×10^3^, 2×10^4^, 2×10^5^, 2×10^6^, and 2×10^7^ CFU/ml for the wild-type strain; 2×10^6^, 2×10^7^, 2×10^8^, 2×10^9^, and 2×10^10^ CFU/ml for the mutant). Female BALB/c mice (6–8 weeks, 5 mice per group) were administered orally with 0.5 ml of bacterial dilutions using a 1.5-in. curved gavage needle with a 2.25-mm ball. After inoculation, mice were weighed and monitored daily for 14 days. Any mouse that lost more than 20% of body weight or that showed signs of extreme morbidity (e.g., shallow breathing and hunched posture) was euthanized by CO_2_ overdose, and scored as a death.

### Assessment of the lethal effect of the induced expression of yncE* on the vaccine bacteria in vitro

Bacteria of the Δ*phoPQ* strain harboring pBAD24-yncE* or the empty pBAD24 vector were serially diluted in PBS. Two microliters of bacterial dilution were inoculated on agar containing 0.1% arabinose. After an overnight-incubation, bacterial growth was observed.

### Evaluation of the lethal effect of the induced YncE* on the vaccine bacteria in the guts of mice

Fresh cultures of the Δ*phoPQ* mutant harboring pBAD24-yncE* were harvested and resuspended in PBS. Female BALB/c mice aged 6–8 weeks (three groups, 10 or 11 mice per group) were orally inoculated with 1×10^9^ CFU of the Δ*phoPQ* mutant harboring pBAD24-yncE* suspended in 0.5 ml PBS, or 0.5 ml PBS alone (as control). At 24 h postvaccination, one group of the immunized mice were orally administered with 0.5 ml of 20% arabinose to induce expression of yncE* in the vaccine bacteria. To quantitate the shedding vaccine bacteria, bacteria were recovered from fecal samples and quantitated for up to 30 days after vaccination (fecal samples were collected everyday in the first week, every 3 days in the remaining weeks). To do that, three to five fecal pellets were collected from each immunized mouse and suspended in PBS at 100 mg/ml. A 100 μl of suspension was plated onto LB agar supplemented with ampicillin to numerate Salmonella. The limit of detection is 10 CFU/g feces.

On the 30th day postvaccination, all mice were orally challenged with 1×10^8^ CFU of the wild-type strain. Mice were weighed and monitored daily for 30 days as described in the assessment of LD_50_.

### Evaluation of the lethal effect of the induced YncE* on the vaccine bacteria in the guts of streptomycin-pretreated mice

BALB/c mice were treated with orally administration of streptomycin (20 mg per mouse) dissolved in sterile water. At 20 h after the streptomycin-treatment, water and food were withdrawn for 4 h before the mice were orally inoculated with 1×10^8^ CFU of the Δ*phoPQ* mutant harboring pBAD24-yncE* (two groups of six mice each) or the empty pBAD24 vector (one group of six mice) suspended in 0.5 ml of PBS.

At 24 h and 48 h postvaccination, the immunized mice were orally given 0.5 ml of 20% arabinose or PBS. Fecal samples were collected and suspended in PBS at 100 mg/ml. After serially diluted, a 100 μl of suspension was plated onto *Salmonella*-*Shigella* agar (SS agar) with ampicillin to quantitate *Salmonella*. For the samples that produced no colony on SS agar with ampicillin, a 100 μl of suspension was inoculated on SS agar without ampicillin to examine the possible existence of *Salmonella*, which escaped from YncE*-killing by losing plasmid or mutation. On the 30th day postvaccination, the tested mice were euthanized by CO_2_ overdose, and their livers, spleens and mesenteric lymph nodes were removed by sterile surgery. The homogenized suspensions of the tissues were plated onto LB agar to enumerate persistent bacteria.

### Flagellin purification

Fresh cultures of *S*. Typhimurium were pelleted, washed and resuspended in PBS. The suspension was shaken violently for 45 min. Bacterial cells were pelleted by centrifugation at 7000 g for 10 min. The supernatant was then centrifuged at 100000 g for 3 h to harvest flagellin. The flagellin protein was dissolved in water, and incubated at 4°C overnight. After a centrifugation at 7000 g, the supernatant was collected. Protein concentration was determined with the BCA assay. The flagellin solution was then preserved at -80°C for future use.

### Determination of IgG levels of anti-flagellin and anti-lipopolysaccharide (anti-LPS) in the serum samples of the immunized mice

Female BALB/c mice (three groups, four mice per group) were immunized as described above. On the 30th day postvaccination, blood samples were collected. Determination of IgG levels of anti-flagellin and anti-LPS in the serum samples was performed with ELISA as described elsewhere [24]. The LPS of *S*. Typhimurium was purchased from Sigma-Aldrich.

### Statistical analysis

LD_50_ was determined by probit analysis. Group means were compared by Kruskal-Wallis test with Dunn’s post-hoc test. Fisher’s exact test was used to analyze survival rates. Values of P < 0.05 were regarded as statistically significant.

## Results

### Construction of the Δ*phoPQ* mutant of *S*. Typhimurium

**Fig 1:**
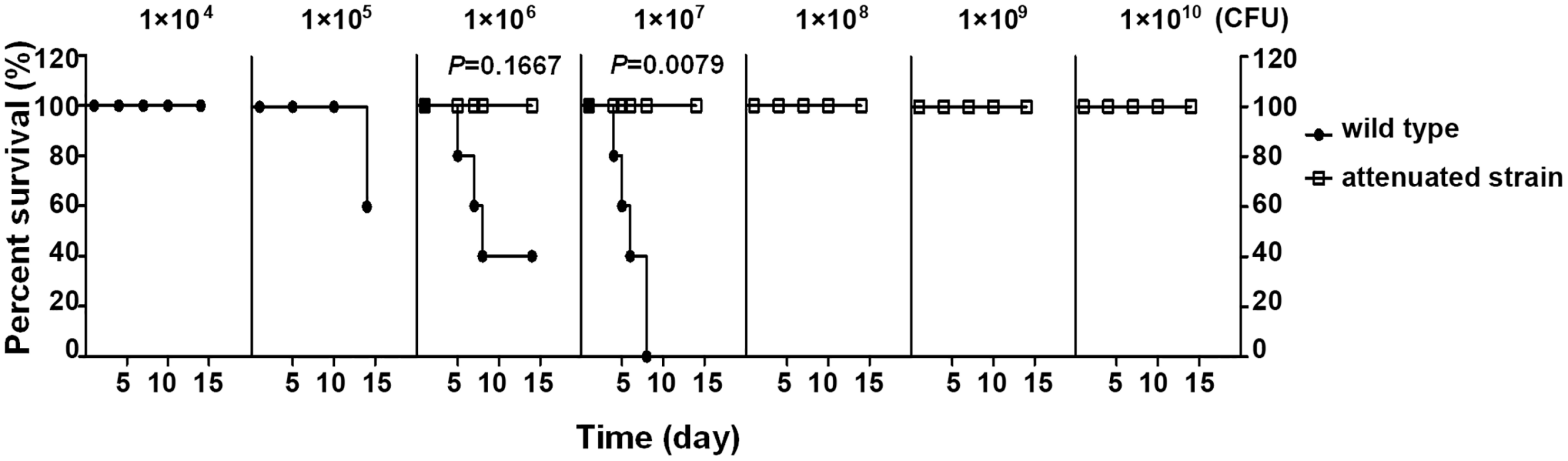
Survival curves of mice challenged with *Salmonella enterica* serovar Typhimurium wild-type or Δ*phoPQ*. Female BALB/c mice aged 6–8 weeks (5 mice per group) were orally challenged with bacteria at indicated doses. Mice were monitored daily for 14 days as described in the section of Materials and methods.

To create an attenuated strain, the *phoPQ* locus was deleted from *S*. Typhimurium CMCC50115 via homologous recombination (S1 Fig). The *phoPQ* locus encodes a two-component regulatory system controlling expression of a large number of genes (>300), many of which are associated with bacterial virulence [25–31]. Therefore, the PhoPQ regulatory system plays a critical role in pathogenicity. Deficiency of *phoPQ* causes reduced bacterial virulence [29, 32, 33]. The *phoPQ* locus has been extensively used in attenuating bacteria [34]. As shown in Fig 1, LD_50_ of the Δ*phoPQ* mutant (>2×10^10^ CFU) was markedly increased compared to that of the wild-type strain (7.58×10^5^ CFU), suggesting that the virulence of the Δ*phoPQ* mutant was largely reduced. The mutant was then used in the subsequent experiment.

### Induced expression of yncE* inhibits the growth of the Δ*phoPQ* mutant in vitro

**Fig 2:**
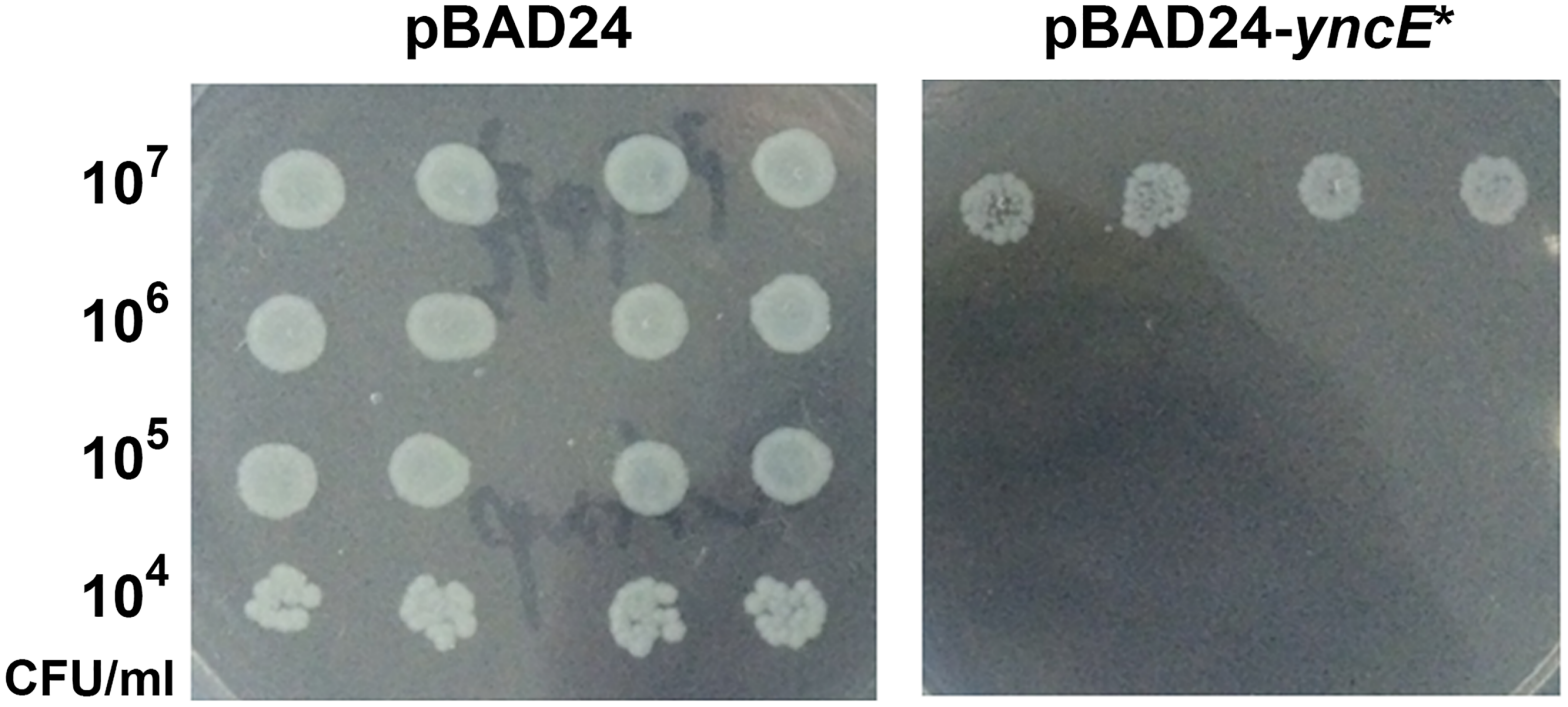
Growth inhibition of *Salmonella enterica* serovar Typhimurium by induced expression of yncE*. Fresh cultures of *S*. Typhimurium harboring pBAD24- yncE* or empty pBAD24 plasmid were serially diluted. Two microliters of dilutions were inoculated and grown on LB agar supplemented with 0.1% of arabinose.

Prior to in vivo experiments, we assessed the lethal effect of the induced YncE* on the growth of the Δ*phoPQ* mutant in vitro. The data show that colony formation of the Δ*phoPQ* mutant harboring pBAD24-yncE* in the presence of 0.1% arabinose was remarkably inhibited compared to the mutant harboring the empty pBAD24 (Fig 2).

### Induced expression of YncE* eliminates the vaccine bacteria in the intestinal tracts of mice

**Fig 3:**
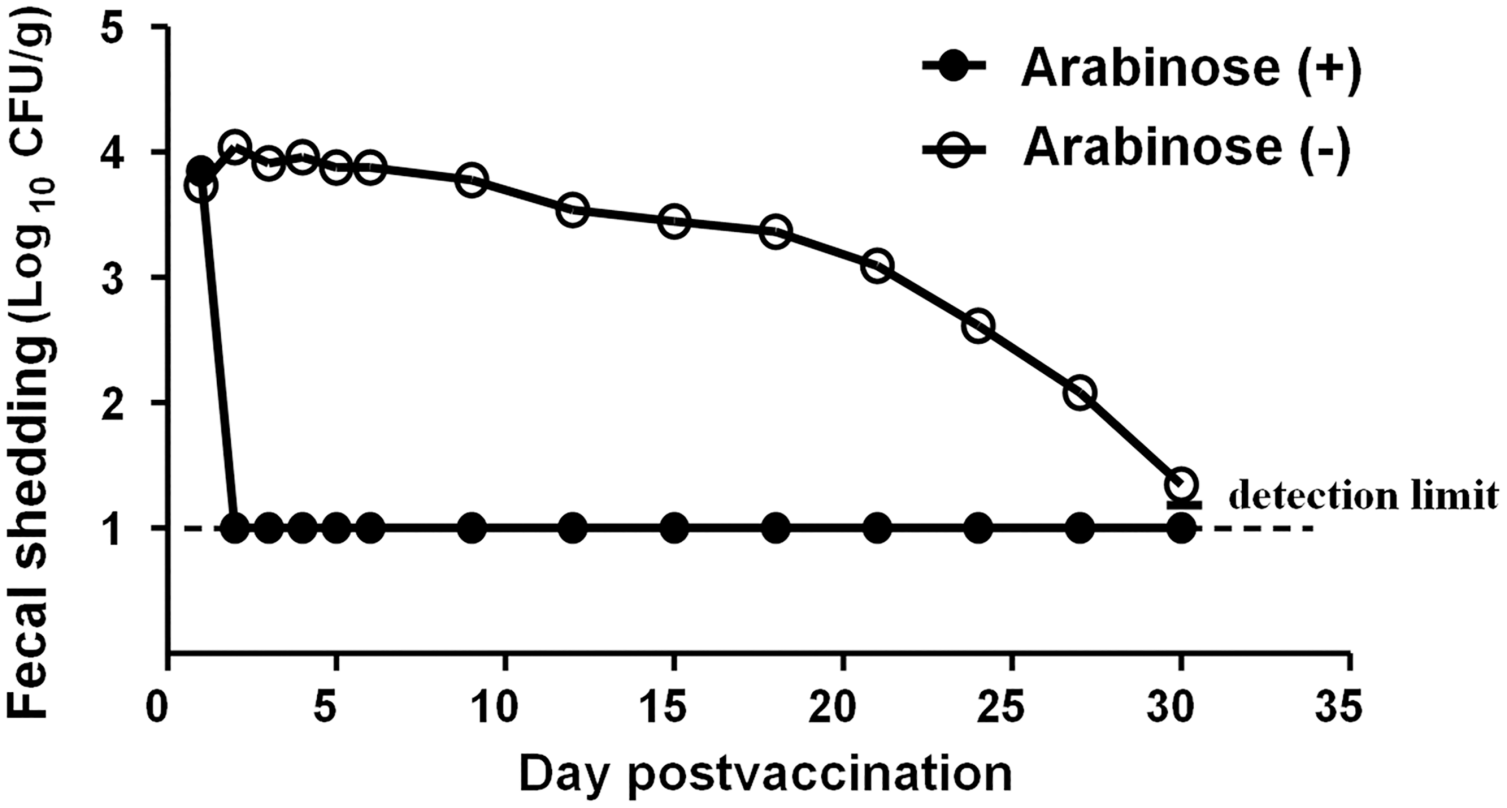
Elimination of the vaccine bacteria in the guts of mice by induced expression of yncE*. Female BABL/c mice (10 or 11 mice per group) were orally immunized with the Δ*phoPQ* mutant of *Salmonella enterica* serovar Typhimurium harboring pBAD24-yncE* (1×10^9^ CFU/mouse). One group of the immunized mice were orally administered with 0.5 ml of 20% arabinose at 24 h postvaccination. The shedding vaccine bacteria in the fecal samples were quantitated by plate cultivation with ampicillin-containing LB agar. The detection limit is 10 CFU/g feces.

Because there are no ampicillin-resistant bacteria in the intestinal tract of the mice used in our animal experiments (confirmed by our preliminary experiments), a simple culture assay with agar supplemented with 100 μg/ml ampicillin was used to quantitate the Δ*phoPQ* mutant bearing the ampicillin-resistant pBAD-derived plasmid. After an oral administration of the Δ*phoPQ* mutant harboring pBAD24-yncE*, fecal samples were collected, and subjected to cultivation and colony counting. A number of live vaccine bacteria were recovered from the fecal samples of the immunized mice without arabinose treatment after inoculation (Fig 3). The amount of the shedding bacteria was slowly decreased. However, even on the 30th day postvaccination, the vaccine bacteria were still detectable in the feces of the immunized mice without arabinose treatment (Fig 3). The results demonstrate that the administrated vaccine bacteria colonized the guts of the immunized mice, and were excreted in the feces for a period of time.

In contrast to the persistent presence of the vaccine bacteria in the guts of the immunized mice without arabinose treatment, the vaccine bacteria were completely eliminated in the guts of the immunized mice with a single oral arabinose treatment, and never recovered from the feces samples (Fig 3). The results show that the induced expression of YncE* eliminated the live vaccine bacteria in the guts of the mice.

**Fig 4:**
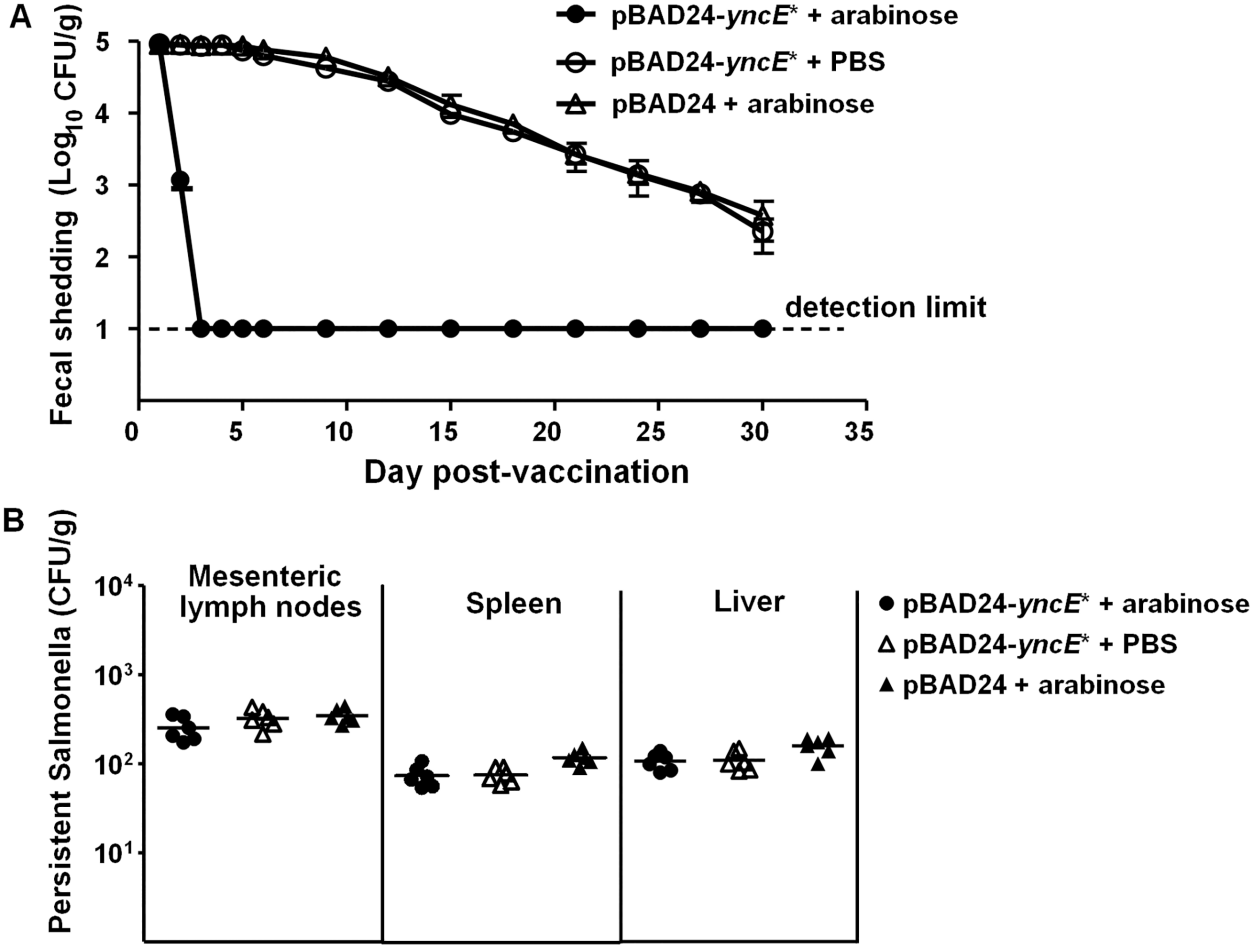
Elimination of the vaccine bacteria in the guts of streptomycin-pretreated mice by induced expression of yncE*. (A) Fecal shedding of the immunized mice. Female BABL/c mice (6 mice per group) were orally pretreated with streptomycin at 24 h prior to immunization with 1×10^8^ CFU of the Δ*phoPQ* mutant harboring pBAD24-yncE* or pBAD24. At 24 h and 48 h postvaccination, the immunized mice were orally administered with 0.5 ml of 20% arabinose or PBS. The shedding vaccine bacteria were recovered on *Salmonella*-*Shigella* agar with ampicillin and quantitated. (B) Persistent bacteria in the internal organs. On the 30th day postvaccination, the tested mice were euthanized, and their livers, spleens and mesenteric lymph nodes were removed by sterile surgery. The homogenized suspensions of the tissues were plated onto LB agar to enumerate persistent bacteria.

Oral treatment with streptomycin can disrupt the indigenous flora of mice, and improves intestinal colonization by exogenous bacteria [35, 36]. Thus, we used streptomycin-pretreated BALB/c mice to further analyze the efficacy of the arabinose-inducible eradication. As expected, higher levels of the vaccine bacteria colonized in the guts of the tested mice with streptomycin treatment (Fig 4A). However, one dose of arabinose was insufficient to remove the vaccine bacteria in the guts. An additional oral arabinose treatment was required for complete clearance of the vaccine bacteria (Fig 4A). For the samples that produced no colony on SS agar with ampicillin, SS agar without ampicillin was used to confirm the results. From the fecal samples that produced no colony on ampicillin-containing SS agar, no vaccine bacteria were recovered on SS agar either. However, the results can not rule out the escape possibility of the vaccine bacteria from the YncE*-killing, which has been observed in vitro. The few escape bacteria were probably eliminated by the multiple hostile factors in the gut, such as low pH, bile, the normal flora, etc.

In contrast to the vaccine bacteria in the guts of mice, the vaccine bacteria resident in internal organs were not significantly impacted by the gavage of arabinose. Amounts of persistent *Salmonella* in the mesenteric lymph nodes, livers and spleens of the immunized mice with arabinose treatment are comparable to those of the mice without arabinose treatment, or the mice immunized with the vaccine bacteria harboring the empty pBAD24 vector (Fig 4B). The data indicate that orally administered arabinose can not reach the bacteria that have penetrated the intestinal mucosa of the tested mice.

### The protective efficacy of vaccination was not significantly altered by the elimination treatment

**Fig 5:**
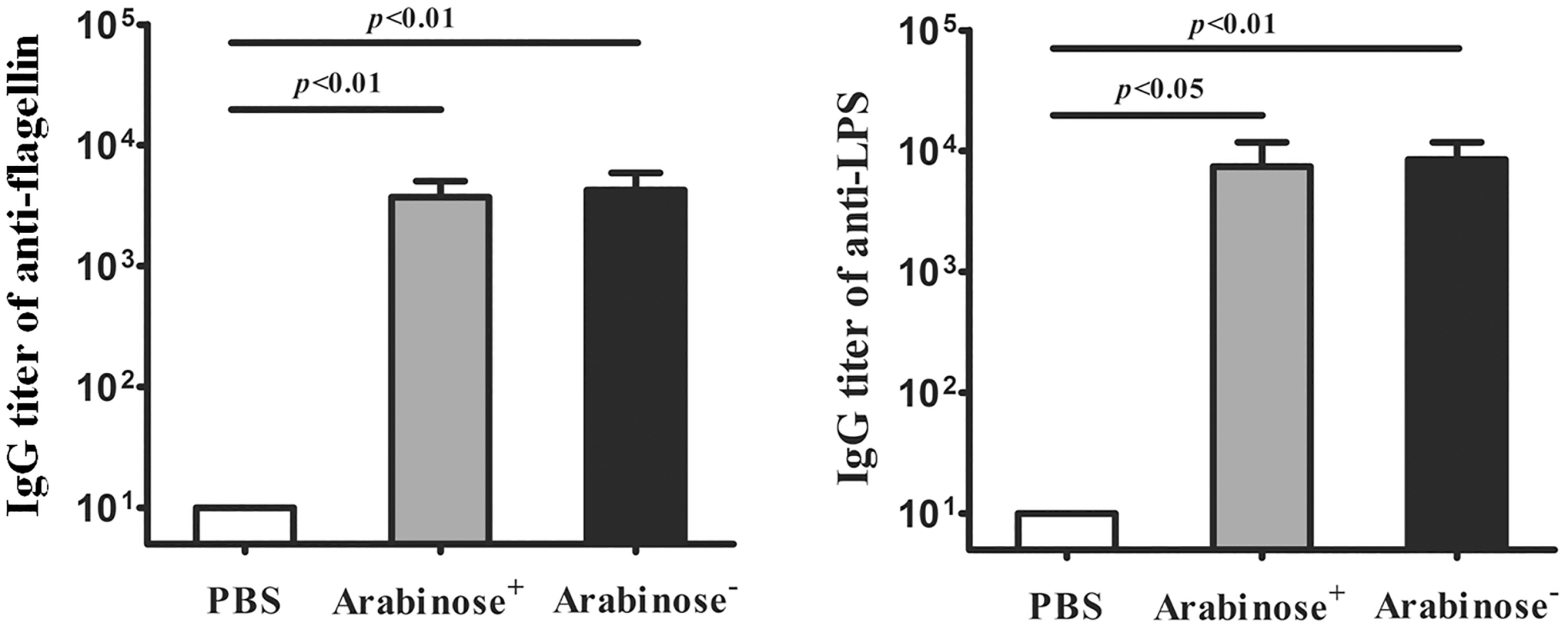
The levels of IgG antibodies in the immunized mice with arabinose treatment are comparable to those in the immunized mice without arabinose treatment. BALB/c mice were orally immunized with the Δ*phoPQ* mutant harboring pBAD24-yncE* at a dose of 1×10^9^ CFU/mouse, or PBS as control. One group of the immunized mice were administered with 0.5 ml of 20% arabinose at 24 h postvaccination. On the 30th day postvaccination, serum samples were harvested. The levels of anti-flagella and anti-LPS IgGs were determined with ELISA.

To explore whether or not the elimination treatment impairs the protective efficacy of the live vaccine, we measured the levels of protective antibodies, the anti-flagellin IgG and the anti-LPS IgG, in the serum samples of the immunized mice. The results show that the IgG levels of anti-flagellin and anti-LPS in the serum samples of the arabinose-treated mice are comparable to those in the serum samples of the mice without arabinose treatment; the mice in both immunized groups, with or without arabinose treatment, produced increased levels of anti-flagellin and anti-LPS IgGs relative to the mice with the mock vaccination (Fig 5, *P*<0.05 or *P*<0.01). The data indicate that the elimination of the vaccine bacteria in the guts of the immunized mice at 24 h postvaccination did not significantly impair the strength of humoral immune responses elicited by flagellin or LPS, two important protective antigens for Salmonella, probably due to the persistent systemic vaccine bacteria.

**Fig 6:**
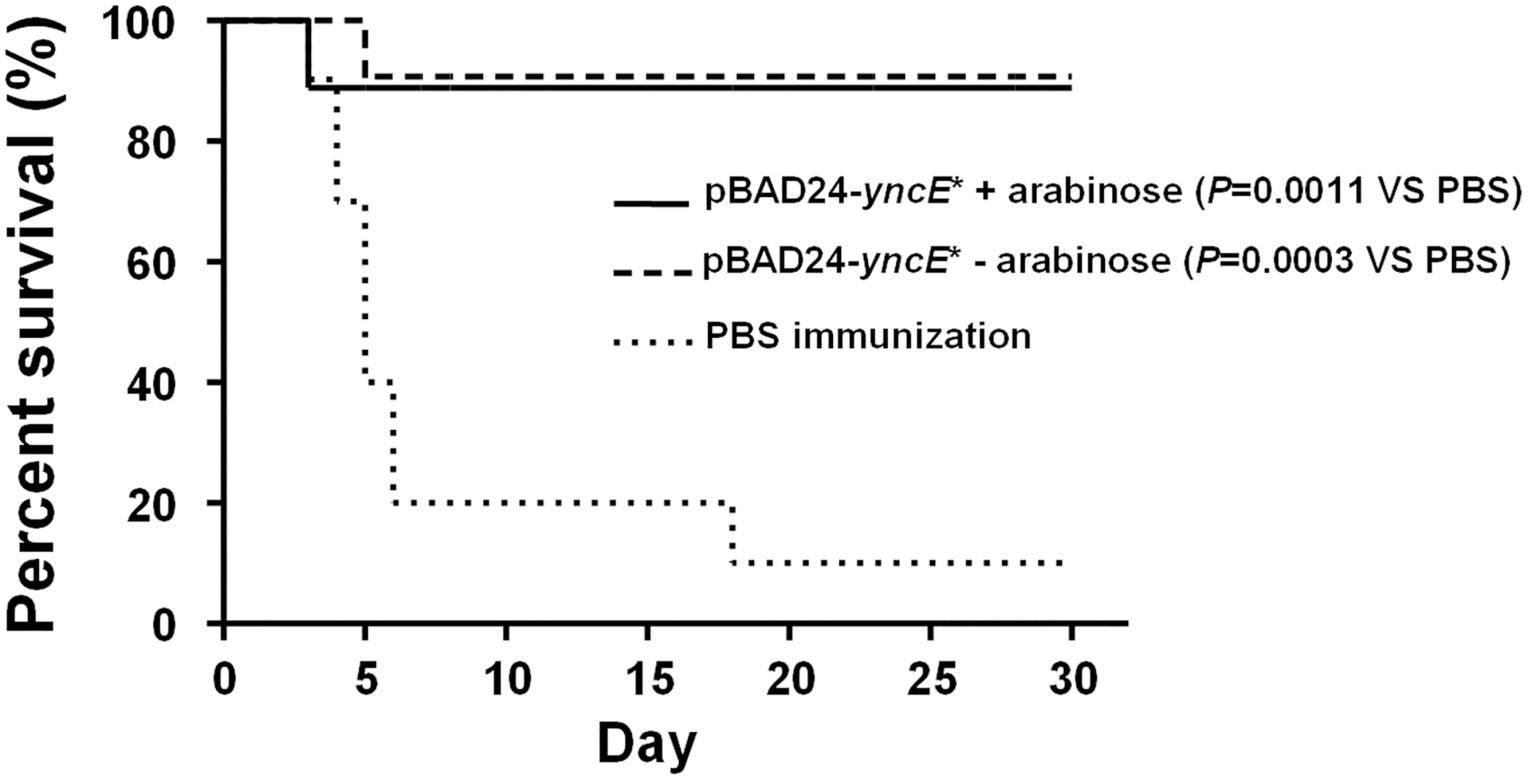
Immunoprotection of orally administered *Salmonella enterica* serovar Typhimurium Δ*phoPQ* with or without a subsequent arabinose-induced bacterial elimination. BALB/c mice were orally immunized with the Δ*phoPQ* mutant harboring pBAD24-yncE* at a dose of 1×10^9^ CFU/mouse, or PBS as control. One group of the immunized mice were administered with 0.5 ml of 20% arabinose at 24 h postvaccination. On the 30th day postvaccination, the tested mice were challenged with the wild-type strain at a dose of 1×10^8^ CFU per mouse. Mice were monitored daily for 30 days as described in the section of Materials and methods.

To assess the level of immunoprotection, on the 30th day postvaccination, the immunized mice were orally challenged with the wild-type strain at a dose of 1×10^8^ CFU/mouse. Consistent with the detection results of the antibody levels, the vaccination with the elimination treatment showed a significant protection against the wild-type challenge compared with the mock immunization (*P*<0.01), at a level comparable to the normal vaccination without the arabinose treatment (Fig 6).

## Discussion

With the rise of synthetic biology and the extensive application of recombinant DNA technology in the past decades, the concerns about the biosafety of genetically modified microbes are growing [37, 38]. One of these concerns is that the microorganisms may escape their intended habitats and cause unpredictable biohazard to the environment [5].

In order to minimize environment release, which can not be completely avoided, to an acceptable level, a large number of studies have focused on the construction of engineered microbes with elaborate designs that allow them to be removed in a non-designated environment [37, 38]. Generally, three strategies are involved in these designs: addiction to an exogenously supplied ligand; self-killing outside of a designated environment; and self-destroying encoded DNA circuitry outside of a designated environment [37]. The use of self-killing switches involves certain toxic genes, whose expression has robust lethal effect on host bacteria. The encoding products of these toxic genes include toxin-antitoxin systems, restriction enzymes, bacteriocins, bacteriophage lytic systems, etc [38].

In the present study, we evaluated the elimination effect of YncE*, a genetic element with bactericidal capability that we determined previously in vitro, on the orally administered *Salmonella* in the guts of immunized mice. The results show that the expression of YncE*, induced by oral addition of arabinose, was able to reduce the vaccine bacteria in the guts of mice to an undetectable level, though multiple treatments were required sometimes. However, the oral arabinose treatment did not affect the systemic vaccine bacteria, probably due to low level of arabinose that entered into circulation by the oral administration. Thus, the eliminating approach is limited to reducing the intestinal luminal vaccine burden, but cannot replace other attenuations that limit systemic bacterial survival and pathogenicity.

For an attenuated vaccine, an optimal balance must be kept between safety and immunogenicity. Excessive attenuation leads to the weakening of immunogenicity. In the case of our experiment, removing the vaccine bacteria too soon may cause reduced immune responses, and consequently decreased efficacy of the vaccine. Since the orally inoculated bacteria of *S*. Typhimurium can develop infection loci in the Peyer’s patches of the small intestine within 3 h [39], we performed the elimination treatment at 24 h postvaccination, a time point that we proposed is sufficient for the vaccine bacteria to penetrate intestinal mucosa and elicit immune responses. Our data show that removal of bacteria from the guts of the immunized mice at this time point did not significantly impair the IgG levels of anti-LPS and anti-flagellin. The immunization by the vaccine bacteria with arabinose treatment effectively protected mice from the wild-type challenge.

## Conclusion

In summary, our data show that the inducible expression of yncE* can effectively reduce the fecal shedding of live vaccine bacteria of *S*. Typhimurium in the mice model. The elimination treatment at 24 h postvaccination does not significantly impact the immunogenicity and efficacy of the vaccine bacteria. This lethal element, yncE*, has an application potential for the biosafety control of oral live bacterial vaccine.

## Supporting information

S1 Fig PCR identification of phoPQ-deletion of *Salmonella enterica* serovar Typhimurium. M: molecular marker; lanes 1 and 3: PCR products of phoPQ-deletion mutant with primers P1 and P4, as well as P5 and P6; lanes 2 and 4: PCR products of the wild-type strain with primers P1 and P4, as well as P5 and P6.
(TIF)

S1 File NC3Rs ARRIVE guidelines checklist. (DOCX)
