# Supplementary figures and images for: Elimination of persistent vaccine bacteria of Salmonella enterica serovar Typhimurium in the guts of immunized mice by inducible expression of truncated YncE
Source: PLoS One. 2017 Jun 19;12(6):e0179649. doi: 10.1371/journal.pone.0179649 (PMC5476278; doi:10.1371/journal.pone.0179649)

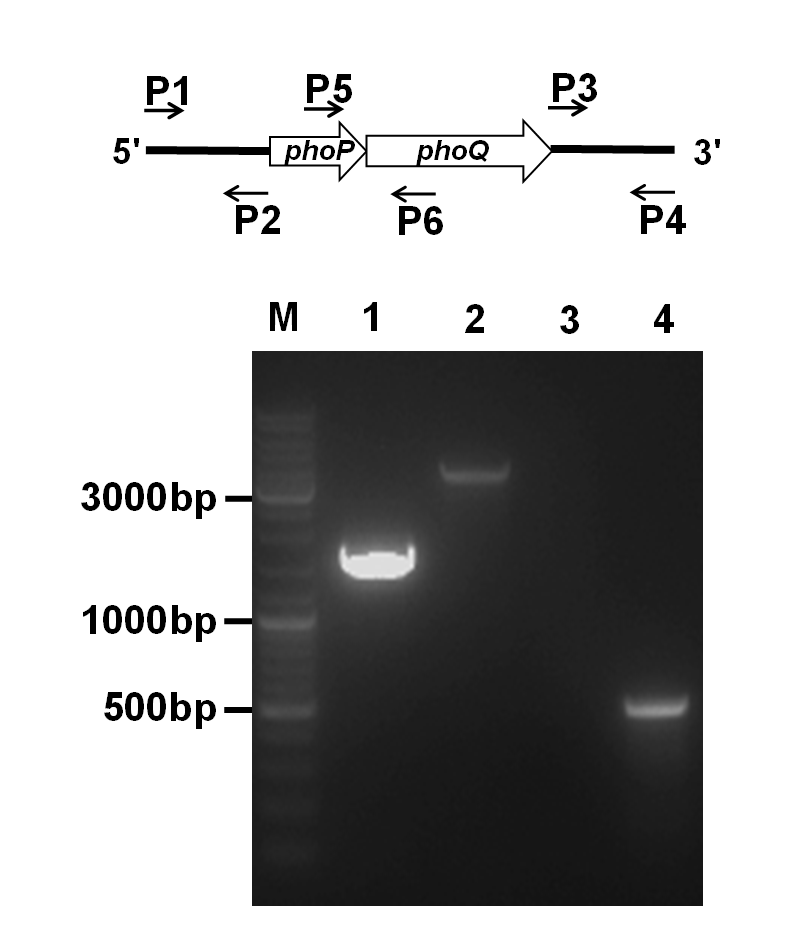

Supplement: S1 Fig — M: molecular marker; lanes 1 and 3: PCR products of phoPQ-deletion mutant with primers P1 and P4, as well as P5 and P6; lanes 2 and 4: PCR products of the wild-type strain with primers P1 and P4, as well as P5 and P6. (TIF) [file pone.0179649.s001.tif]
