# Supplementary material for: Elimination of persistent vaccine bacteria of Salmonella enterica serovar Typhimurium in the guts of immunized mice by inducible expression of truncated YncE
Source: PLoS One. 2017 Jun 19;12(6):e0179649. doi: 10.1371/journal.pone.0179649 (PMC5476278; doi:10.1371/journal.pone.0179649)

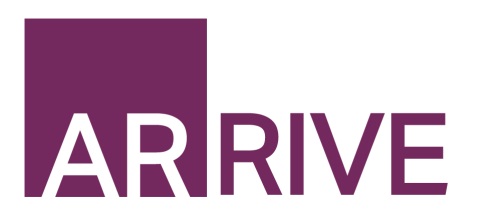


The ARRIVE Guidelines Checklist

Animal Research: Reporting In Vivo Experiments

Carol Kilkenny^1^, William J Browne^2^, Innes C Cuthill^3^, Michael Emerson^4^ and Douglas G Altman^5^

*^1^The National Centre for the Replacement, Refinement and Reduction of Animals in Research, London, UK, ^2^School of Veterinary Science, University of Bristol, Bristol, UK, ^3^School of Biological Sciences, University of Bristol, Bristol, UK, ^4^National Heart and Lung Institute, Imperial College London, UK, ^5^Centre for Statistics in Medicine, University of Oxford, Oxford, UK.*

|  | ITEM | RECOMMENDATION | Section/ Paragraph |
| --- | --- | --- | --- |
| 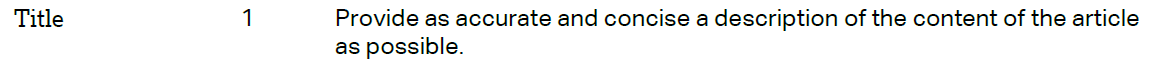 | | | Title |
| 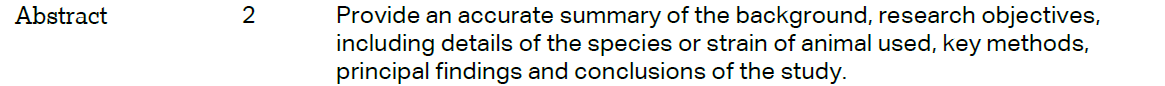 | | | Abstract |
| INTRODUCTION | | |  |
| 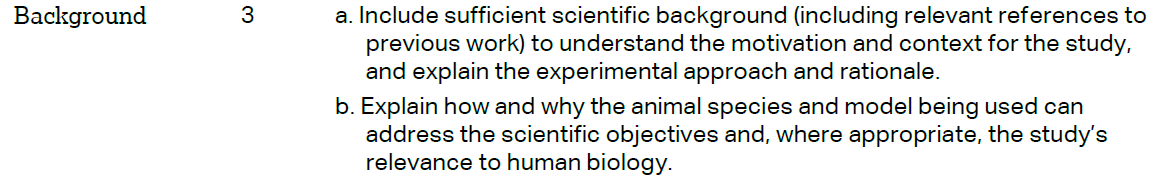 | | | Paragraphs 1-3 |
| 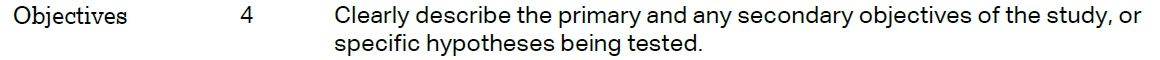 | | | Paragraph 3 |
| METHODS | | |  |
| 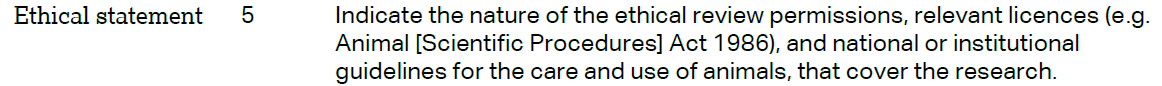 | | | Paragraph 1 |
| 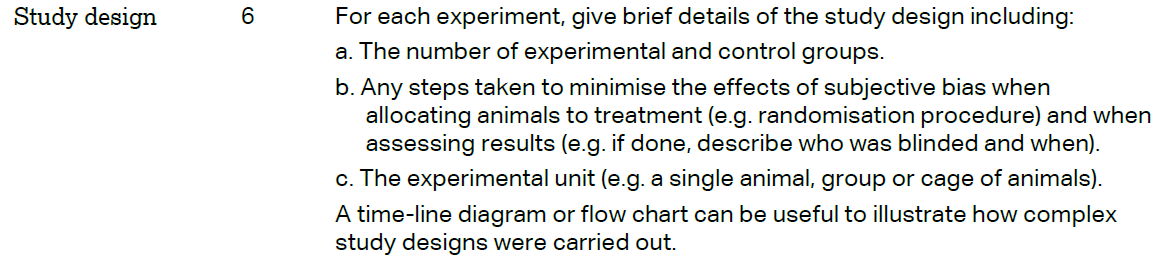 | | | Paragraphs 4,6,7,9  Paragraphs 4,6,7,9  Paragraphs 4,6,7,9 |
| 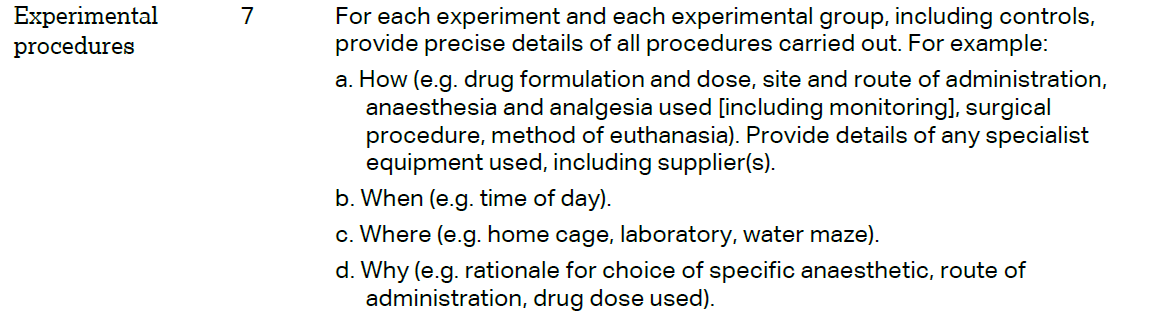 | | | Paragraphs 4,6,7,9 |
| 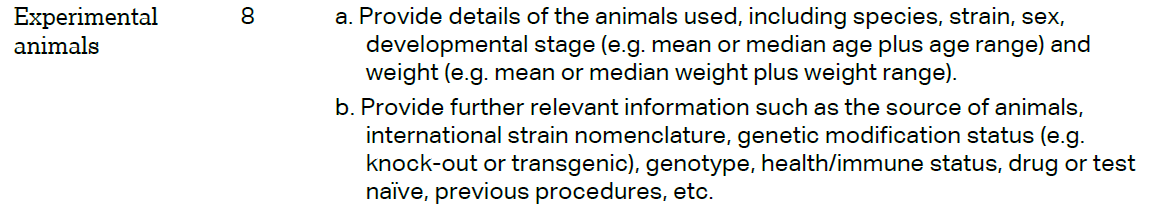 | | | Paragraphs 4,6,7,9 |

The ARRIVE guidelines. Originally published in *PLoS Biology*, June 2010^1^

| 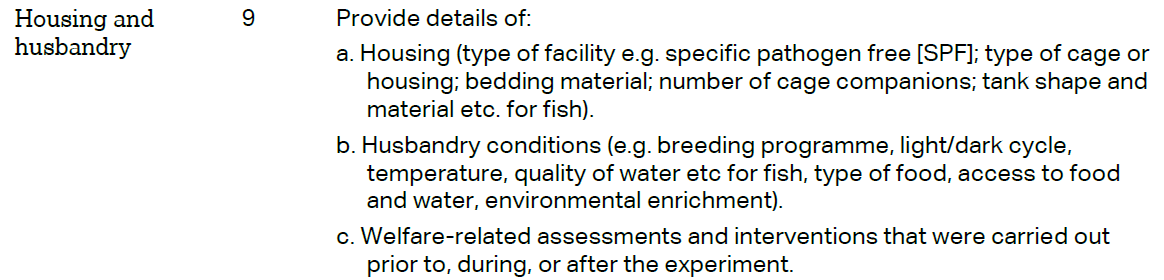 | Paragraph 1 | |
| --- | --- | --- |
| 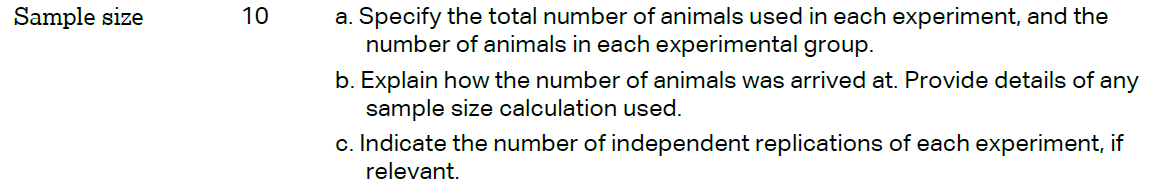 | Paragraphs 4,6,7,9 | |
| 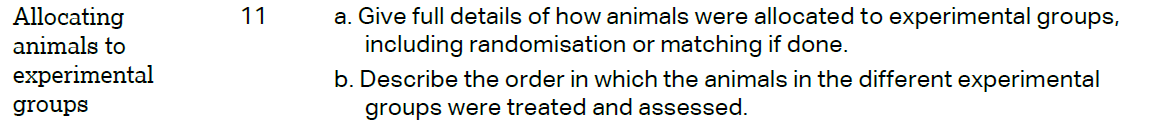 | Paragraphs 4,6,7,9 | |
| 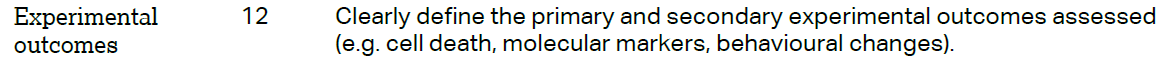 | Paragraphs 4,6,7,9 | |
| 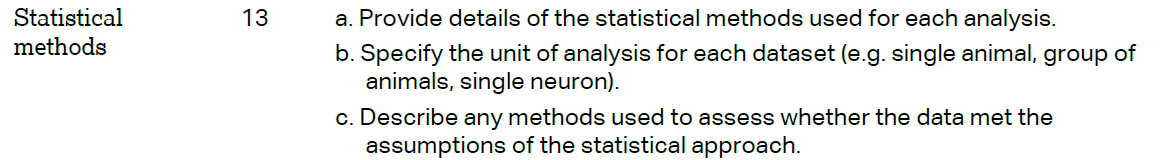 | Paragraph 10 | |
| RESULTS |  | |
| 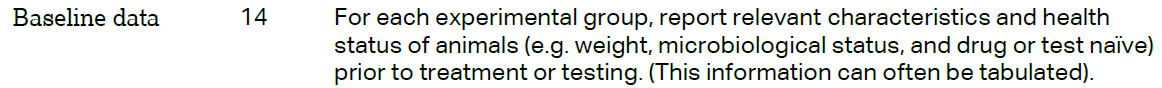 | Methods Paragraphs 4,6,7,9 | |
| 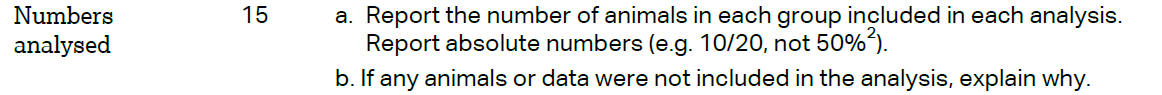 | Methods Paragraphs 4,6,7,9 | |
| 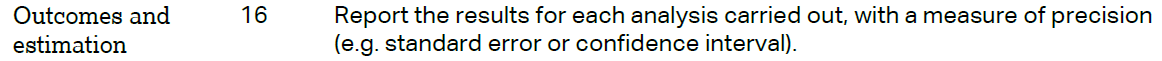 | Paragraphs 3,4 | |
| 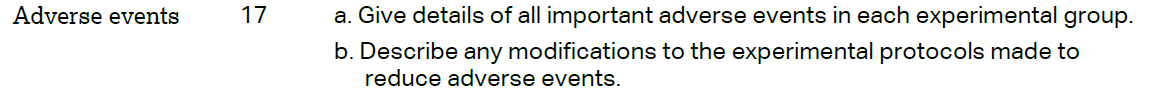 | Methods  Paragraphs 4 | |
| DISCUSSION |  | |
| 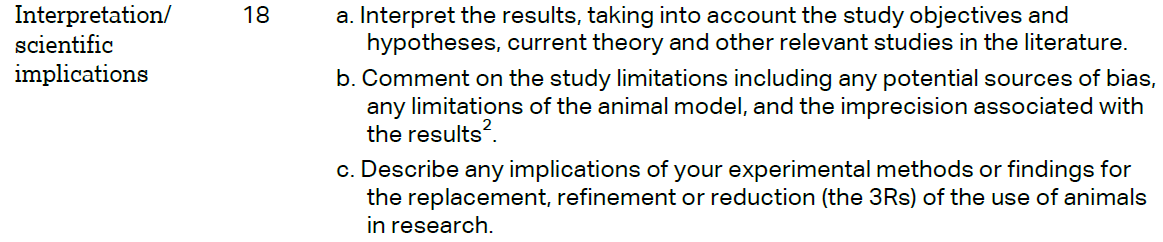 | Throughout  Paragraph 3,4 | |
| 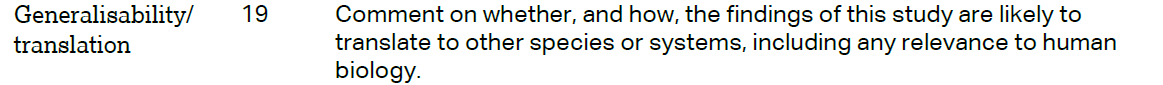 | Conclusion | |
| 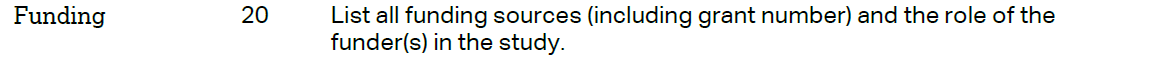 | Acknowledgement |  |


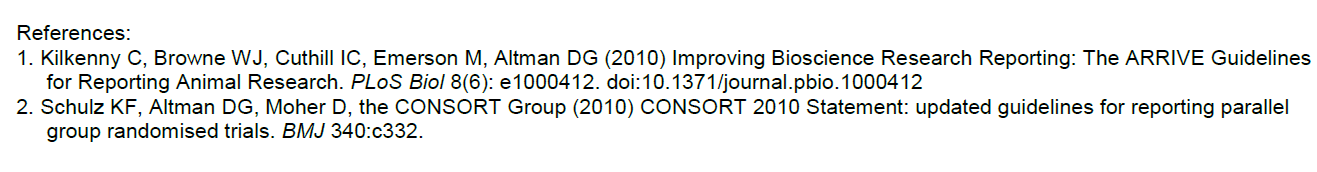

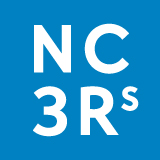

Supplement: S1 File — (DOCX) [file pone.0179649.s002.docx]
